# Population mobility : spatial spillover effect of government health expenditure in China

**DOI:** 10.1080/16549716.2024.2319952

**Published:** 2024-03-11

**Authors:** Simin Wan, Mengying Wang

**Affiliations:** School of Public Finance & Public Administration, Jiangxi University of Finance and Economics, Nanchang, China

**Keywords:** Government health expenditure (GHE), universal health coverage (UHC), dynamic spatial durbin model, population mobility, spatial spillover effect

## Abstract

**Background:**

Since the 20th century, pursuing Universal Health Coverage (UHC) has emerged as an important developmental objective in numerous countries and across the global health community. With the intricate ramifications of population mobility (PM), the government faces a mounting imperative to judiciously deploy health expenditure to realise UHC effectively.

**Objective:**

This study aimed to construct a comprehensive UHC index for China, assess the spatial effects of Government Health Expenditure (GHE) on UHC, and explore the moderating effects of PM on this association.

**Method:**

A Dynamic Spatial Durbin Model (DSDM) was employed to investigate the influence of the GHE on UHC. Therefore, we tested the moderating effect of PM.

**Results:**

In the short-term, the GHE negatively impacted local UHC. However, it enhanced the UHC in neighbouring regions. Over the long term, GHE improved local UHC but decreased UHC in neighbouring regions. In the short-term, when the PM exceeded 1.42, the GHE increased the local UHC. Over the long term, when the PM exceeded 1.107, the GHE impeded local UHC. If the PM exceeded 0.91 in the long term, the GHE promoted UHC in neighbouring regions. The results of this study offer a partial explanation of GHE decisions and behaviours.

**Conclusions:**

To enhance UHC, a viable strategy involves augmenting vertical transfer payments from the central government to local governments. Local governments should institute healthcare systems tailored to the urban scale and developmental stages, with due consideration for PM. Optimising the information disclosure mechanism is also a worthwhile endeavour.

## Introduction

Despite China’s status as a developing nation with a population of 1.4 billion, over the past decade, it has successfully expanded its foundational healthcare and safety infrastructure to encompass more than 95% of its population [1]. Through a United Nations resolution, China has recognised Universal Health Coverage (UHC) as a pivotal global health objective and has made considerable strides towards achieving UHC in alignment with the Sustainable Development Goals (SDG). However, the path towards attaining UHC is not without challenges. At the level of UHC advancement, effective monitoring and a comprehensive grasp of the quantifiable details enabled by such monitoring are indispensable for advancing the UHC agenda. Previous studies have consistently focussed on constructing indicators of UHC [2,3]. Some scholars have proposed indicators for evaluating UHC in China based on micro-regional surveys [4,5]. However, few indicators have been used to fully evaluate UHC in various regions of China. This study aims to construct a set of indicators for UHC to assess its progress in China. These indicators were formulated based on standards set by the World Health Organization (WHO) and the World Bank, considering China’s specific circumstances and data availability.

Dr. Margaret Chan, WHO’s director-general, described UHC as ‘the single most powerful concept that public health has to offer’ [6]. Previous studies have mainly assessed the performance of Government Health Expenditure (GHE) based on UHC [7,8]. However, these studies have comparatively limited discussions on the spatial effects of GHE on UHC and have not adequately addressed factors such as population mobility (PM), regional disparities, economic development, and other pertinent characteristics. The government should fund UHC in public health services [9,10]. Local governments endowed with information advantages can tailor the provision of diverse public goods to meet residents’ preferences [11]. The effective supply of public goods can be achieved if residents can move between communities and ‘vote with their feet.’ As the most populous country in the world, China has experienced significant population movement. By the end of 2021, China’s floating population reached 385 million [12], making PM an undeniable social development phenomenon. PM significantly influences government resource allocation and public health and exhibits discernible spatial spillover effects [13]. Therefore, exploring the impact of GHE on UHC from the PM perspective holds considerable practical significance.

**Figure 1.**
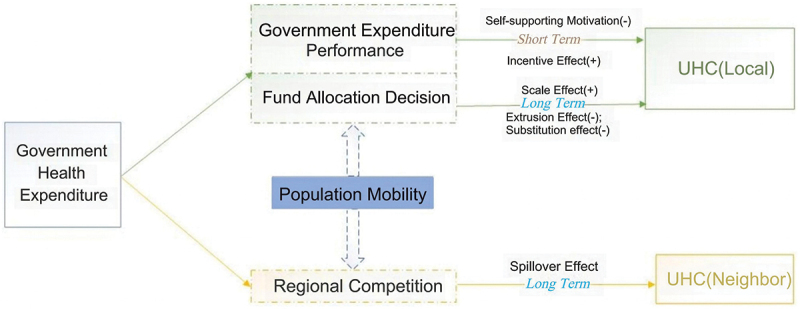
Influence mechanism of GHE on UHC.

In summary, this study undertook the following endeavours. First, we constructed a comprehensive indicator system for UHC in China. Second, departing from conventional approaches for assessing the association between the GHE and UHC, we employed a Dynamic Spatial Durbin Model (DSDM) to examine the spatial effects of the GHE on UHC. Considering health expenditure objectives and performance, a GHE typically yields diverse effects. The objectives of GHE primarily involve augmenting infrastructure development, such as expanding the number and scale of hospitals. The performance of GHE is predominantly assessed by examining health-quality indicators, including incidence rates. As shown in Figure 1, governmental inclination towards prioritising economic growth targets in the short-term may engender self-support motivation. Simultaneously, the influence of demonstration effects from neighbouring regions may introduce incentive effects. In the long term, GHE may exhibit scale effects. PM is a crucial factor that influences government decisions concerning health investments. This may affect the allocation of government resources and competition among neighbouring regions, leading to demonstration or extrusion effects. Hence, this study scrutinised whether PM moderated the impact of the GHE on UHC, thereby enhancing the comprehensiveness and scientific rigour of the conclusions.

## Method

### Data description

Based on data availability, this study compiled empirical testing data from 2004 to 2018, encompassing 31 provinces (municipalities and autonomous regions) in China. All original data sources included authoritative publications such as the ‘China Service Industry Statistical Yearbook,’ ‘China Finance Yearbook,’ ‘China Financial Yearbook,’ ‘China Statistical Yearbook,’ ‘China Agricultural Statistical Yearbook,’ and ‘China Health Statistics Yearbook.’ All the data used in this study were obtained from the National Bureau of Statistics of China. The final sample size of this study amounted to 465. A spatial weight matrix was utilised to generate spatial data to facilitate spatial regression analysis. We designated the geographical spatial data of n regions as ${\left\{x_{i}\right\}}_{i=1}^{n}$, where the subscript i represents region i. If we denote the distance between region i and region j as $w_{\mathrm{ij}}$, the spatial weight matrix can be defined as

(1) $$W=\left(\begin{array}{ccc} w_{11} & \cdots & w_{1j}\\ \vdots & \ddots & \vdots \\ w_{i1} & \cdots & w_{\mathrm{ij}} \end{array}\right)$$

The elements on the main diagonal, $w_{11}=\ldots =w_{\mathrm{ij}}=0$, signify the distances within the same region, defined as 0. In this study, a spatial distance weight matrix was employed; the specific formula is as follows:

(2) $$w_{\mathrm{ij}}=1\;/\;d_{\mathrm{ij}}(i\neq j)$$

The $d_{\mathrm{ij}}$ denotes the geographical distance between the capitals of 31 provinces (municipalities and autonomous regions) of China. To facilitate the investigation of dynamic effects in subsequent analyses, a one-period lag was applied to the UHC and GHE variables.

### Variables for the study

This study employs UHC as the dependent variable and GHE as the primary independent variable. The UHC index, chosen as a proxy variable for UHC, is a comprehensive indicator. A detailed introduction to the UHC index is provided in the section titled ‘Construction of UHC index’. We selected per-capita GHE as a proxy variable for GHE. Drawing upon existing studies on the factors influencing UHC, we incorporate the following control variables: (1) technological advancement (TA) [14]. We used the number of invention patents owned by the pharmaceutical manufacturing industry per pharmaceutical manufacturing enterprise to indicate the level of local technological advancement. (2) Population mobility (PM). Some studies examined the relationship between immigration and UHC [15]. This study investigated PM in China. PM is defined as the ratio between the resident and registered populations. (3) Per capita gross domestic product (PGDP) [2]. (4) Per capita private health expenditure (PHE) [16]. (5) Aging [17]. We adopted the elderly dependency ratio to measure the degree of aging, which is the ratio of the number of people aged 65 years and above to the number of people aged 15–65 years. (6) Human capital (HC) [18]. Human capital accumulation is achieved primarily through investment in education. Therefore, the average number of years of education in each region was selected as the measure of human capital.

### Construction of UHC index

When constructing a UHC index, it is imperative to diversify the selection of tracking indicators and their corresponding weights to ensure comparability. This enables the state to effectively evaluate and monitor national health development [19]. Based on the WHO and the World Bank UHC monitoring framework, and considering insights from existing studies, we opted for the following four dimensions to assess the progress of UHC in China: (1) promotion/prevention, (2) treatment, (3) service and accessibility, and (4) financial protection [20,21] (Table 1). Eleven of our UHC indicators (antenatal care coverage (ACC), examination before marriage (EBM), percentage of the population using improved drinking water sources (PID), percentage of the population using improved sanitation facilities (PDS), gynaecological examination (GE), fatality rate of tuberculosis (FRTB), incidence rate of whooping cough (IRWC), incidence rate of tetanus in newborn (IRTN), health institutions per 1000 (HI), health per 1000 (HP), beds per 1000 (BD)) were proposed based on the WHO and World Bank monitoring framework [22]. Furthermore, two additional indicators (delivery in hospital (DIH) and proportion of urban health insurance (UHIC)) were added, based on the UHC indicators of Meng et al. [23]. Table 2 presents the descriptive statistics for the UHC indicators.

**Table 1.** Universal health coverage index.

1st indices	2nd indices	3rd indices	Abbreviation	Rationale	Weight	Positive indicator/Negative indicator
Universal Health Coverage	Promotionb /prevention	Antenatal care coverage	ACC	Proposed in the World Bank and WHO Monitoring Framework.	0.05	P
		Examination Before Marriage	EBM	Proposed as a characterization indicator for the “reproductive” component of the World Bank and WHO Monitoring Framework.	0.05	P
		Percentage of the population using improved drinking water sources	PID	Proposed in the World Bank and WHO Monitoring Framework; Included in the World Bank and WHO Monitoring Report.	0.05	P
		Percentage of the population using improved sanitation facilities	PDS	Proposed in the World Bank and WHO Monitoring Framework; Included in the World Bank and WHO Monitoring Report.	0.05	P
		Gynecological Examination	GE	Proposed as a characterization indicator for the “reproductive” component of the World Bank and WHO Monitoring Framework.	0.05	P
	Treatment	Delivery in Hospital	DIH	Proposed for inclusion in Meng et al.’s health service indicators in China.	0.0625	P
		Incidence Rate of Whooping Cough	IRWC	Proposed as an alternative indicator for Child immunization (DTP3) in the World Bank and WHO Monitoring Frameworks.	0.0625	N
		Incidence Rate of Tetanus in Newborn	IRTN		0.0625	N
		Fatality Rate of Tuberculosis	FRTB	Proposed as an alternative indicator for TB effective treatment (TB) in the World Bank and WHO Monitoring Frameworks.	0.0625	N
	Service and Accessibility	Health Institutions Per 1000	HI	Proposed as an important supplementary indicator of service and accessibility.	0.0667	P
		Health per 1000	HP	Proposed in the World Bank and WHO Monitoring Framework.	0.0667	P
		Beds per 1000	BD	Proposed in the World Bank and WHO Monitoring Framework.	0.0667	P
	Financial protection	Proportion of Urban Health Insurance	UHIC	Proposed for inclusion in Meng et al.’s financial protection indicators in China.	0.25	P

**Table 2.** Descriptive statistics of universal health coverage indicators.

Variables	Max	Min	Mean	Std. Dev.
ACC	100.00	27.21	82.30	14.21
EBM	100.00	0.18	34.21	32.09
PID	100.00	26.61	68.55	18.79
PDS	99.80	18.20	64.39	18.72
GE	165.62	9.70	56.38	26.62
DIH	100.00	26.74	92.71	13.00
IRWC	7.05	0.00	0.37	0.79
IRTN	10.35	0.00	0.22	0.92
FRTB	2.49	0.00	0.32	0.29
HI	21.79	1.24	5.49	3.55
HP	15.46	2.00	5.06	1.92
BD	7.55	1.48	3.96	1.45
UHIC	0.76	0.02	0.18	0.13

It is crucial to emphasise that in selecting the 13 UHC indicators, we applied normalisation to standardise them. Specifically, ACC, EBM, PID, PDS, GE, DIH, HI, HP, BD, and UHIC were positive indicators. IRWC, IRTN, and FRTB were negative indicators. The normalisation formula is as follows:

(3) $$X_{\mathrm{positive}}=(x-\min\;(x))/(\max\;(x)-\min\;(x))$$

(4) $$X_{\mathrm{negative}}=(\max\;(x)-x)/(\max\;(x)-\min\;(x))$$

Following the construction framework recommended by the WHO and World Bank, the UHC index was calculated as the geometric mean of the tracking indicators [20]. In particular, we assigned a weight of 0.25 to the second-level indices, representing an average of four indicators that track the progress of prevention, treatment, service, and financial protection. Subsequently, we calculated the average weights of the third-level indices within the same second-level index (Table 1). Utilising the geometric mean instead of the arithmetic mean is preferred because it ensures equal representation of various services at the same coverage level rather than favouring the improvement of certain services at the expense of others [22].

**Figure 2.**
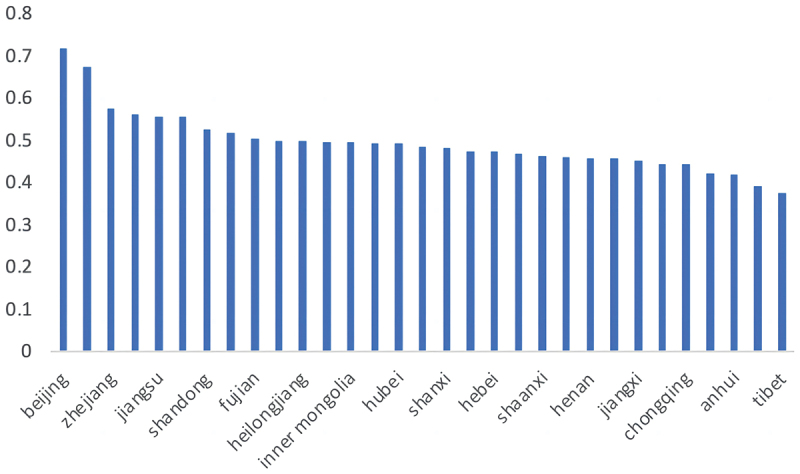
Ranking of UHC index in China.

Figure 2 illustrates the regional hierarchy of China’s UHC index from 2004 to 2018. The graphical representation reveals Beijing as the leading region in UHC, followed by Shanghai and Jiangsu. Among these regions, Beijing achieved the highest level at 0.71, whereas Tibet recorded the lowest level at 0.38, underscoring the noteworthy disparity between the two regions.

### Data analysis

First, a descriptive statistical analysis of the variables was performed. Second, Moran’s index, commonly employed for measuring spatial autocorrelation [24], was utilised by applying the spatial weight matrix to analyse the spatial correlation of the UHC index in China. Moran’s index is calculated as follows:

(5) $$I=\frac{\sum_{i=1}^{n}\sum_{j=1}^{n}w_{\mathrm{ij}}\left(x_{i}-\overset{ˉ}{x}\right)\left(x_{j}-\overset{ˉ}{x}\right)}{s^{2}\sum_{i=1}^{n}\sum_{j=1}^{n}w_{\mathrm{ij}}}$$

{s^2} = {{\mathop \sum \nolimits_{i = 1}^n {{\left({{x_i} - \mathop \bar \bar x\limits^ } \right)}^2}} \over n} represents the sample variance, $\sum_{i=1}^{n}\sum_{j=1}^{n}w_{\mathrm{ij}}$ is the sum of the spatial domains of the weight matrix values. A Moran’s index greater than 0, within the range of −1 to 1, signifies a positive spatial correlation. Conversely, a value less than 0 indicates a negative correlation, whereas an index close to 0 implies a weak spatial correlation. The local Moran’s index further dissects Moran’s index by regions. Moran’s index is a correlation coefficient between observed values and spatial lags.

Finally, we investigated the spatial effects of the GHE on UHC. In contrast to the Static Spatial Durbin Model (SSDM), DSDM incorporates a temporal dimension and considers the spatial autocorrelation of the dependent variable. The parameter estimation of the independent variables and error terms in the DSDM remain unaffected by omitted variables. Furthermore, the DSDM encompasses spatially and temporally lagged variables for independent and dependent variables. Therefore, this study can effectively estimate the direct and indirect effects (spillover effects) of the GHE on UHC along with the long- and short-term effects of the GHE on UHC [25]. To compute these effects, we applied the ‘derivative-seeking approach’ proposed by Lesage and Pace [26] combined with the formulation provided in equation (6). Consequently, we used the DSDM and conducted fitting using the Maximum Likelihood Estimation (MLE) [27]. The formula for the DSDM is as follows:

(6) $$\mathrm{UH}C_{\mathrm{it}}=\alpha_{0}+\mathrm{\tau UH}C_{\mathrm{it}-1}+\rho \;W\mathrm{UH}C_{\mathrm{it}}+\varphi \;W\mathrm{UH}C_{\mathrm{it}-1}+\beta_{1}\mathrm{GH}E_{\mathrm{it}}+\beta_{2}\mathrm{GH}E_{\mathrm{it}}*PM_{\mathrm{it}}+\beta_{n}\mathrm{Contro}l_{\mathrm{it}}+\theta \;W\mathrm{GH}E_{\mathrm{it}}+\mu_{i}+\lambda_{t}+\varepsilon_{\mathrm{it}}$$

In equation (6), $\mathrm{UH}C_{\mathrm{it}}$ represents UHC, the dependent variable. $\alpha_{0}$ is a constant. $\mathrm{UH}C_{\mathrm{it}-1}$ is the lagged value of UHC. In addition, we applied smoothing to the UHC by taking the logarithm. $\mathrm{GH}E_{\mathrm{it}}$ represents GHE, which is the primary independent variable. W is the spatial weight matrix. $PM_{\mathrm{it}}$ represents the population mobility. $\mathrm{GH}E_{\mathrm{it}}*PM_{\mathrm{it}}$ is the interaction term. $\mathrm{Contro}l_{\mathrm{it}}$ represents control variables. $\beta_{1}$ represents the coefficient of $\mathrm{GH}E_{\mathrm{it}}$ and $\beta_{2}$ is the coefficient of the interaction term. θ represents the spatial regression coefficient of $\mathrm{GH}E_{\mathrm{it}}$. τ represents the time lag coefficient of $\mathrm{UH}C_{\mathrm{it}}$, ρ represents the spatial regression coefficient of $\mathrm{UH}C_{\mathrm{it}}$, φ represents the spatio-temporal lag coefficient of $\mathrm{UH}C_{\mathrm{it}}$. $\mu_{i}$ represents spatial-fixed effects, and $\lambda_{t}$ represents time-fixed effects. $\varepsilon_{\mathrm{it}}$ represents spatial autocorrelation error term.

## Results

### Regional spatial correlation of UHC index

**Figure 3.**
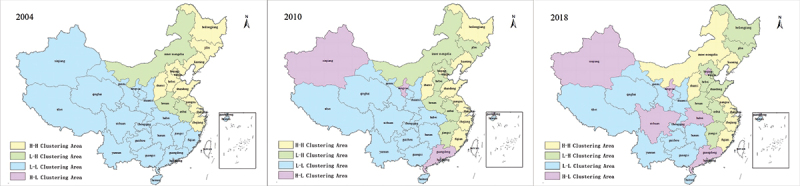
The spatial correlation of UHC in 2004, 2010, and 2018.

We computed the regional Moran’s I value for the UHC data from 2004 to 2018. Figure 3 illustrates the findings for 2004, 2010, and 2018 (Figure 3).

Based on Figure 3, it is apparent that the UHC in Western China are primarily clustered within the low-low spatial arrangement. Conversely, high-high clusters were predominantly concentrated in Eastern China. UHC distribution pattern persisted throughout the period from 2004 to 2018. Furthermore, a significant proportion of the areas in Central China fell within the low-high clustering area. Notably, the provinces encompassing the high-low clustering area underwent substantial fluctuations. We stratified the regional spatial correlation of UHC into two distinct stages from 2004 to 2018.

During the initial stage, from 2004 to 2009, the clustered areas with high UHC were mainly located in eastern and northeastern China, including Beijing, Tianjin, Jiangsu, Shanghai, Zhejiang, Liaoning, and Jilin. Inner Mongolia and some of Central China were situated in the low-high clustering area. In contrast, Western China and the remaining part of Central China fell into the low-low clustering area, as exemplified by Xinjiang and Tibet. In the second stage, from 2010 to 2018, UHC’s high and high-low clustering areas showed significant expansion. Inner Mongolia has transitioned from a low-high clustering area to a high-high clustering area. Concurrently, Xinjiang, Ningxia, Hunan, Hubei, and Sichuan gradually shifted towards high-low clustering areas, indicating the proximity of provinces with high UHC to those with low UHC. Following implementing the new medical system reform in 2009, certain provinces experienced a rapid increase in UHC, leading to higher UHC levels than neighbouring regions.

### Spatial econometric model analysis

Firstly, Table 3 reports the descriptive statistics of the variables in this study.

**Table 3.** Descriptive statistics of variables.

Variable type	Variable name	Obs	Mean	Std. Dev.	Min	Max
Dependent variable	UHC	465	0.49	0.11	0.21	0.77
Independent variable	GHE	465	5.87	4.68	0.29	31.08
Control variables	TA	465	2.52	3.64	0	38.63
	PM	465	1.03	0.16	0.80	1.69
	PGDP	465	3.8357	2.5495	0.4316	14.0211
	PHE	465	7.40	4.83	0.90	29.00
	Aging	465	0.13	0.03	0.07	0.23
	HC	465	8.61	1.22	3.74	12.56

Table 4 presents the results of the spatial regression analysis. In the short term, the direct impact of GHE on UHC was significantly negative ($\beta_{\mathrm{short}\_\mathrm{direct}}$ = -0.047, p <0.01). This means that the local GHE hindered the improvement of local UHC in the short term. Simultaneously, there existed an indirect positive effect of GHE on UHC ($\beta_{\mathrm{short}\_\mathrm{indirect}}$ = 0.050, p <0.01). This means that, in the short term, local GHE plays a positive role in UHC in neighbouring regions.

**Table 4.** Spatial spillover effect decomposition of UHC with DSDM.

Indicator Name	Short-Term Effect			Long-Term Effect		
	Direct	Indirect	Total	Direct	Indirect	Total
GHE	−0.047***	0.050***	0.003	0.144***	−0.142***	0.002
	(0.00407)	(0.00664)	(0.00491)	(0.015)	(0.0158)	(0.00305)
GHE*Population Mobility	0.033***	0.009	0.042***	−0.130***	0.156***	0.026***
	(0.0033)	(0.00608)	(0.00464)	(0.0135)	(0.0141)	(0.00288)
Rho	3.720***					
	(0.053)					
Year	yes					
ID	yes					
Control	yes					
_cons	yes					
R-squared	0.207					

*, **, and *** indicate significance at the 10%, 5%, and 1% levels, respectively.

In the long run, the direct effect of GHE on UHC was significantly positive ($\beta_{\mathrm{long}\_\mathrm{direct}}$ = 0.144, p <0.01). This indicates that in the long term, local GHE promoted the advancement of local UHC. The indirect effect of GHE on UHC was significantly negative ($\beta_{\mathrm{long}\_\mathrm{indirect}}$=-0.142, p <0.01). This indicates that, in the long run, local GHE will adversely impact UHC in neighbouring regions.

Based on the spatial regression results (Table 4) and computational outcomes, it was ascertained that under the influence of PM, the GHE had a significant impact on UHC, with the existence of critical thresholds. Concerning the short-term direct effect, when the PM exceeded the threshold of 1.42, local GHE exhibited a positive influence on local UHC ($\beta_{\mathrm{short}\_\mathrm{direct}}+\beta_{\mathrm{pm}\_\mathrm{short}\_\mathrm{direct}}*\mathrm{PM}$>0,p<0.01). For the long-term direct effect, when PM exceeded the threshold of 1.107, local GHE manifested a detrimental effect on local UHC ($\beta_{\mathrm{long}\_\mathrm{direct}}+\beta_{\mathrm{pm}\_\mathrm{long}\_\mathrm{direct}}*\mathrm{PM}>0$, p<0.01). Regarding the short-term indirect effect, PM did not play a significant role in the impact of GHE on UHC ($\beta_{\mathrm{pm}\_\mathrm{short}\_\mathrm{indirect}}=0.009$,p>0.1). For the long-term indirect effect, when PM exceeded the threshold of 0.91, the impact of GHE on UHC was significantly positive ($\beta_{\mathrm{long}\_\mathrm{indirect}}+\beta_{\mathrm{pm}\_\mathrm{long}\_\mathrm{indirect}}*\mathrm{PM}$>0,p<0.01). This suggests that local GHE exhibits a demonstrative effect on the UHC in neighbouring regions.

## Discussion

We summarise the spatial regression results and provide possible explanations. First, the GHE had no overall significant impact on UHC. However, the short-term direct, short-term indirect, long-term direct, and long-term indirect effects were significant. (1) Short-term direct effects. In the short-term, governments often implement measures to reduce spending on public services such as health and education to achieve economic growth objectives [28], representing self-supporting motivation. This practice has detrimental implications for the UHC. (2) Short-term indirect effects. A positive indirect effect on UHC existed in neighbouring regions. A plausible rationale for this outcome is that a GHE in a particular region can promote economic development [29]. When a local region obtains economic benefits by increasing GHE, it may play a demonstration (incentive) role in neighbouring regions, prompting them to increase the scale of GHE and consequently enhance UHC. (3) Long-term direct effects. In the long term, the government will continue to increase investments in healthcare, foster economies of scale, and expand the provision of healthcare services, ultimately playing a positive role in promoting local UHC. (4) Long-term indirect effects. From the previous analysis, it was evident that local GHE had a positive guiding effect on neighbouring regions in the short term. However, local GHE may negatively impact neighbouring regions in the long term because of the existence of a fiscal ‘ceiling’ [28]. Under the influence of the short-term indirect effect, neighbouring regions may adopt proactive policies for the GHE. Over time, mounting fiscal pressure could overwhelm neighbouring regions, reducing the government’s capacity in neighbouring regions to provide essential medical equipment, personnel, and medications. Consequently, there may be a dearth of adequate infrastructure and a decline in the quality of healthcare services, ultimately impeding UHC achievement.

Second, when PM was considered, the total, direct, and indirect effects of the GHE on UHC were significant. (1) When PM > 1.42, the local GHE has a significant positive impact on the local UHC in the short term. The regional PM influences local governments’ expenditure preferences and allocation decisions [30]. In regions with low PM, individuals choose to reside primarily because of familial connections, natural surroundings, and other factors, rather than relying on basic public services. Consequently, enhancing UHC may not be the primary government’s focus in these areas. Governments tend to allocate their budgets to administrative expenses in the short term, thus neglecting public service expenditures and, by extension, UHC. For cities with large PM, the convenience of public services is considered a priority [31]. As a larger population migrates to a region, individuals become more cognizant of government expenditure effectiveness and demand appropriate public services, thereby driving UHC forward [32]. Thus, a high population count amplifies the short-term impact of the GHE on UHC. (2) When PM > 1.107, the local GHE exhibits a significant negative effect on the local UHC in the long term. In the long term, the government remains focussed on delivering essential public services, and increasing specialisation leads to a rise in marginal output, establishing a scale effect that enhances UHC. However, the endowment of the various production factors in a city is limited. Public and private hospitals are important components of the healthcare system and important choices for patients seeking medical treatment [33]. To ensure UHC, it is imperative that both public and private hospitals collaborate. GHE primarily targets public hospitals [34]. Over time, sustained government investment influences the operations of private hospitals. On the one hand, the endowment of various production factors in the region is limited, and government investment will attract medical professionals from private hospitals to public hospitals. On the other hand, the government’s price subsidy for public medical services has reduced residents’ demand for quasi-public goods in private hospitals, and the operation of private hospitals is hindered or even bankrupt [34]. That is, the extrusion effect of public hospitals on private hospitals caused by local GHE may directly reduce local UHC. (3) When PM > 0.91, the local GHE had a significant positive effect on UHC in neighbouring regions in the long term. In theory, considering PM, a long-term spillover effect is anticipated in neighbouring regions [13]. In the long term, the government’s provision of UHC, a crucial public good, significantly influences the interregional flow of labour, technology, and capital. Consequently, a competitive dynamic emerged between governments at the regional level. Competition among governments can incentivise them to serve residents better and allocate resources more efficiently [35]. In regions experiencing an influx of population, the deliberate increase in GHE serves as an example, fostering the advancement of UHC in neighbouring regions.

## Conclusions

Based on the findings of this study, we found that the GHE exerted dynamic spatial effects on UHC and that the magnitude of PM played a central moderating role. To advance UHC, we recommend optimising the information disclosure mechanism and bolstering public engagement in allocating and utilising government public health funds. The central government is advised to employ vertical transfer payments as an incentive mechanism for local governments to encourage active investments in public health and ensure prudent fund utilisation. Simultaneously, local governments should be attentive to the impact of PM and endeavour to refine and enhance healthcare systems tailored to the size and developmental stage of their respective cities.

## Supplementary Material

235235649 Supplementary files.pdf

## Data deposition

The data that support this study are available in public databases at https://www.stats.gov.cn/english/

## Paper context

- The authors confirmed the dynamic spatial impact of Government Health Expenditure on Universal Health Coverage.
- Population mobility played a moderating role in the effect of Government Health Expenditure on Universal Health Coverage.
- The national government should implement vertical transfer, and local governments should institute healthcare systems commensurate with the scale and developmental stage to promote Universal Health Coverage and improve public health.
