# Supplementary material for: Population mobility : spatial spillover effect of government health expenditure in China
Source: Glob Health Action. 2024 Mar 11;17(1):2319952. doi: 10.1080/16549716.2024.2319952 (PMC10930106; doi:10.1080/16549716.2024.2319952)
Supplement: 235235649 Supplementary files.pdf [file ZGHA_A_2319952_SM2424.pdf]

Supplementary file 1. Spatial weight matrix values (rounded to two decimal places)

	_W1	_W2	_W3	_W4	_W5	_W6	_W7	_W8	_W9	_W10	_W11	_W12	_W13	_W14	_W15	_W16	_W17	_W18	_W19	_W20	_W21	_W22	_W23	_W24	_W25	_W26	_W27	_W28	_W29	_W30	_W31
**SWMImpo**	0	0.17	0.28	0.05	0.04	0.04	0.02	0.02	0.02	0.02	0.02	0.02	0.01	0.01	0.05	0.03	0.02	0.01	0.01	0.01	0.01	0.02	0.01	0.01	0.01	0.01	0.02	0.02	0.01	0.02	0.01
**No**	0.18	0	0.21	0.05	0.04	0.04	0.03	0.02	0.02	0.03	0.02	0.03	0.02	0.02	0.07	0.03	0.02	0.02	0.01	0.01	0.01	0.02	0.01	0.01	0.01	0.01	0.03	0.02	0.01	0.02	0.01
**Yes**	0.27	0.19	0	0.05	0.04	0.03	0.02	0.02	0.02	0.03	0.02	0.02	0.01	0.01	0.05	0.03	0.02	0.01	0.01	0.01	0.01	0.02	0.01	0.01	0.01	0.01	0.02	0.01	0.01	0.02	0.01
**Yes**	0.06	0.06	0.07	0	0.04	0.03	0.02	0.02	0.03	0.03	0.02	0.04	0.02	0.03	0.05	0.07	0.04	0.03	0.02	0.02	0.01	0.03	0.02	0.02	0.02	0.01	0.07	0.03	0.02	0.05	0.01
**Yes**	0.09	0.07	0.08	0.06	0	0.05	0.04	0.04	0.03	0.03	0.02	0.03	0.02	0.02	0.04	0.04	0.03	0.02	0.02	0.02	0.01	0.02	0.02	0.02	0.02	0.02	0.04	0.03	0.02	0.04	0.02
**Yes**	0.07	0.08	0.07	0.04	0.05	0	0.10	0.05	0.03	0.04	0.03	0.03	0.02	0.02	0.06	0.03	0.03	0.02	0.02	0.02	0.01	0.02	0.02	0.02	0.01	0.01	0.03	0.02	0.02	0.03	0.01
**Yes**	0.05	0.05	0.05	0.04	0.05	0.12	0	0.10	0.03	0.04	0.03	0.03	0.02	0.02	0.04	0.03	0.03	0.02	0.02	0.02	0.01	0.02	0.02	0.02	0.02	0.01	0.03	0.02	0.02	0.03	0.01
**Yes**	0.05	0.05	0.05	0.04	0.05	0.07	0.13	0	0.03	0.03	0.03	0.03	0.02	0.02	0.04	0.03	0.03	0.02	0.02	0.02	0.02	0.02	0.02	0.02	0.02	0.02	0.03	0.02	0.02	0.03	0.02
**Yes**	0.03	0.03	0.03	0.03	0.02	0.03	0.02	0.02	0	0.11	0.12	0.08	0.05	0.05	0.05	0.04	0.04	0.03	0.03	0.02	0.02	0.02	0.02	0.02	0.02	0.01	0.03	0.02	0.01	0.02	0.01
**Yes**	0.03	0.04	0.04	0.03	0.02	0.03	0.02	0.02	0.10	0	0.07	0.11	0.04	0.04	0.07	0.05	0.04	0.03	0.02	0.02	0.01	0.02	0.02	0.02	0.01	0.01	0.03	0.02	0.01	0.02	0.01
**Yes**	0.02	0.03	0.03	0.03	0.02	0.02	0.02	0.01	0.12	0.07	0	0.08	0.08	0.07	0.04	0.04	0.04	0.04	0.03	0.02	0.02	0.03	0.02	0.02	0.02	0.01	0.02	0.02	0.01	0.02	0.01
**Yes**	0.03	0.03	0.03	0.03	0.02	0.02	0.02	0.01	0.07	0.11	0.07	0	0.04	0.05	0.05	0.07	0.06	0.04	0.03	0.02	0.01	0.03	0.02	0.02	0.02	0.01	0.03	0.02	0.01	0.02	0.01
**Yes**	0.02	0.02	0.02	0.02	0.02	0.02	0.02	0.01	0.05	0.04	0.08	0.05	0	0.12	0.03	0.04	0.04	0.05	0.06	0.04	0.03	0.03	0.02	0.03	0.02	0.01	0.03	0.02	0.01	0.02	0.01
**Yes**	0.02	0.02	0.02	0.03	0.02	0.02	0.01	0.01	0.04	0.04	0.06	0.06	0.10	0	0.03	0.04	0.06	0.07	0.06	0.04	0.02	0.04	0.02	0.03	0.02	0.01	0.03	0.02	0.01	0.02	0.01
**Yes**	0.06	0.08	0.07	0.05	0.03	0.04	0.03	0.02	0.04	0.07	0.03	0.06	0.02	0.03	0	0.06	0.04	0.02	0.02	0.02	0.01	0.02	0.02	0.02	0.01	0.01	0.03	0.02	0.01	0.03	0.01
**Yes**	0.04	0.04	0.04	0.06	0.02	0.02	0.02	0.01	0.03	0.05	0.03	0.07	0.03	0.04	0.05	0	0.08	0.04	0.02	0.02	0.01	0.04	0.02	0.03	0.02	0.01	0.06	0.02	0.02	0.04	0.01
**Yes**	0.03	0.03	0.03	0.04	0.02	0.02	0.01	0.01	0.03	0.04	0.04	0.06	0.04	0.06	0.03	0.08	0	0.07	0.03	0.03	0.02	0.06	0.03	0.04	0.02	0.01	0.05	0.02	0.02	0.03	0.01
**Yes**	0.02	0.02	0.02	0.03	0.02	0.02	0.01	0.01	0.03	0.03	0.04	0.04	0.05	0.08	0.03	0.04	0.08	0	0.06	0.06	0.02	0.06	0.03	0.06	0.03	0.01	0.03	0.02	0.02	0.03	0.01
**Yes**	0.02	0.02	0.02	0.02	0.02	0.02	0.01	0.01	0.03	0.03	0.04	0.04	0.07	0.07	0.03	0.03	0.04	0.08	0	0.08	0.05	0.04	0.03	0.05	0.03	0.01	0.03	0.02	0.02	0.02	0.01
**Yes**	0.02	0.02	0.02	0.02	0.02	0.02	0.01	0.01	0.03	0.03	0.03	0.03	0.04	0.05	0.02	0.03	0.04	0.07	0.08	0	0.04	0.05	0.04	0.10	0.05	0.02	0.03	0.02	0.02	0.02	0.01
**Yes**	0.02	0.02	0.02	0.03	0.02	0.02	0.02	0.02	0.03	0.03	0.04	0.03	0.05	0.05	0.03	0.03	0.04	0.05	0.08	0.07	0	0.04	0.03	0.05	0.04	0.02	0.03	0.02	0.02	0.03	0.02
**Yes**	0.02	0.02	0.02	0.03	0.02	0.02	0.01	0.01	0.02	0.03	0.03	0.03	0.03	0.04	0.03	0.04	0.07	0.07	0.03	0.04	0.02	0	0.06	0.08	0.04	0.02	0.05	0.03	0.02	0.04	0.01

**Yes**	0.02	0.02	0.02	0.03	0.02	0.02	0.02	0.01	0.02	0.02	0.02	0.03	0.02	0.03	0.02	0.04	0.04	0.04	0.03	0.04	0.02	0.08	0	0.07	0.06	0.03	0.05	0.05	0.05	0.05	0.02
**Yes**	0.02	0.02	0.02	0.03	0.02	0.02	0.01	0.01	0.02	0.02	0.03	0.03	0.03	0.04	0.02	0.03	0.05	0.07	0.04	0.09	0.03	0.09	0.06	0	0.06	0.02	0.04	0.02	0.02	0.03	0.01
**Yes**	0.02	0.02	0.02	0.03	0.02	0.02	0.02	0.01	0.02	0.02	0.03	0.03	0.03	0.03	0.02	0.03	0.04	0.04	0.04	0.06	0.03	0.06	0.07	0.08	0	0.03	0.04	0.03	0.04	0.03	0.02
**Yes**	0.03	0.03	0.03	0.03	0.03	0.02	0.02	0.02	0.02	0.02	0.02	0.03	0.02	0.03	0.03	0.03	0.03	0.03	0.03	0.03	0.02	0.04	0.05	0.04	0.05	0	0.04	0.05	0.08	0.04	0.06
**Yes**	0.03	0.03	0.04	0.07	0.03	0.02	0.02	0.01	0.02	0.03	0.02	0.03	0.02	0.03	0.04	0.07	0.05	0.03	0.02	0.02	0.01	0.05	0.04	0.03	0.02	0.02	0	0.04	0.03	0.09	0.01
**Yes**	0.03	0.03	0.03	0.04	0.03	0.02	0.02	0.02	0.02	0.02	0.02	0.03	0.02	0.02	0.03	0.03	0.03	0.03	0.02	0.02	0.02	0.04	0.05	0.03	0.03	0.03	0.05	0	0.09	0.09	0.03
**Yes**	0.03	0.03	0.03	0.03	0.03	0.02	0.02	0.02	0.02	0.02	0.02	0.02	0.02	0.02	0.03	0.03	0.03	0.03	0.02	0.03	0.02	0.04	0.06	0.04	0.04	0.06	0.04	0.11	0	0.05	0.04
**Yes**	0.04	0.03	0.04	0.06	0.03	0.02	0.02	0.02	0.02	0.03	0.02	0.03	0.02	0.02	0.03	0.04	0.04	0.03	0.02	0.02	0.01	0.04	0.04	0.03	0.02	0.02	0.10	0.07	0.04	0	0.02
**Yes**	0.03	0.03	0.03	0.03	0.03	0.03	0.02	0.02	0.02	0.03	0.02	0.03	0.02	0.03	0.03	0.03	0.03	0.03	0.02	0.03	0.02	0.03	0.04	0.03	0.03	0.07	0.04	0.06	0.07	0.04	0

Supplementary file 2. Code

```
cap log c
clear all
**china
cd "D:/meng"

use "D:/meng/dili.dta", clear
save "dili.dta",replace
spatwmat using dili, name(W) st

use "D:/meng/data2.dta", clear
    keep if year==2004
    spatgsa UHC , weights(W) moran geary twotail
    spatlsa UHC , weights(W) moran graph(moran) sy(n)

use "D:/meng/data2.dta", clear
  keep if year==2005
    spatgsa UHC , weights(W) moran geary twotail
    spatlsa UHC , weights(W) moran graph(moran) sy(n)

use "D:/meng/data2.dta", clear
    keep if year==2006
    spatgsa UHC , weights(W) moran geary twotail
    spatlsa UHC , weights(W) moran graph(moran) sy(n)

use "D:/meng/data2.dta", clear
    keep if year==2007
    spatgsa UHC , weights(W) moran geary twotail
    spatlsa UHC , weights(W) moran graph(moran) sy(n)

use "D:/meng/data2.dta", clear
    keep if year==2008
    spatgsa UHC , weights(W) moran geary twotail
    spatlsa UHC , weights(W) moran graph(moran) sy(n)

use "D:/meng/data2.dta", clear
    keep if year==2009
    spatgsa UHC , weights(W) moran geary twotail
    spatlsa UHC, weights(W) moran graph(moran) sy(n)

use "D:/meng/data2.dta", clear
    keep if year==2010
    spatgsa UHC , weights(W) moran geary twotail
```

```
    spatlsa UHC , weights(W) moran graph(moran) sy(n)

use "D:/meng/data2.dta", clear
    keep if year==2011
    spatgsa UHC , weights(W) moran geary twotail
    spatlsa UHC , weights(W) moran graph(moran) sy(n)

use "D:/meng/data2.dta", clear
    keep if year==2012
    spatgsa UHC , weights(W) moran geary twotail
    spatlsa UHC , weights(W) moran graph(moran) sy(n)

use "D:/meng/data2.dta", clear
    keep if year==2013
    spatgsa UHC , weights(W) moran geary twotail
    spatlsa UHC , weights(W) moran graph(moran) sy(n)

use "D:/meng/data2.dta", clear
    keep if year==2014
    spatgsa UHC , weights(W) moran geary twotail
    spatlsa UHC , weights(W) moran graph(moran) sy(n)

use "D:/meng/data2.dta", clear
    keep if year==2015
    spatgsa UHC , weights(W) moran geary twotail
    spatlsa UHC , weights(W) moran graph(moran) sy(n)

use "D:/meng/data2.dta", clear
    keep if year==2016
    spatgsa UHC , weights(W) moran geary twotail
    spatlsa UHC , weights(W) moran graph(moran) sy(n)

use "D:/meng/data2.dta", clear
    keep if year==2017
    spatgsa UHC , weights(W) moran geary twotail
    spatlsa UHC , weights(W) moran graph(moran) sy(n)

use "D:/meng/data2.dta", clear
    keep if year==2018
    spatgsa UHC , weights(W) moran geary twotail
    spatlsa UHC , weights(W) moran graph(moran) sy(n)
```

```
clear all
use "D:/meng/data2.dta", clear
xtset provcd year
gen PPM=GHE*PM
local x " UHC GHE TA PM HC PHE Aging PGDP PPM"
    tabstat `x', s(mean sd min p25 p50 p75 max) ///
                        format(%6.4f) c(s)
reg UHC GHE TA PM HC PGDP PHE Aging PPM
xtreg UHC GHE TA PM HC PGDP PHE Aging PPM,fe
est store eq_fe
xtreg UHC GHE TA PM HC PGDP PHE Aging PPM,re
est store eq_re
hausman eq_fe eq_re
ssc install xtscc
xtscc UHC GHE TA PM HC PGDP PHE Aging PPM,fe

***LM test****
use "D:/meng/data2.dta", clear
ssc install spwmatrix
spwmatrix import using"weightd.dta",wname(W) dta xtw(15)
generate lnUHC=ln(UHC)
gen PPM=GHE*PM
reg y lnUHC GHE TA PM PGDP PHE Aging HC PPM
spatdiag, weights(W)

use "D:/meng/dili.dta", clear
save "dili.dta",replace
  spatwmat using dili, name(W) st
use "D:/meng/quyu.dta", clear
sort province
save "quyu.dta",replace
use "D:/meng/datahe.dta", clear
sort province
merge province using quyu.dta

use "D:/meng/dataghe.dta", clear
generate lnUHC=ln(UHC)
gen PPM=GHE*PM
xtset provcd year
xsmle lnUHC GHE TA PM PGDP PHE Aging HC PPM, model(sar) wmat(W) fe
est store eq_1
xsmle lnUHC GHE TA PM PGDP PHE Aging HC PPM, model(sar) wmat(W) re
est store eq_2
xsmle lnUHC GHE TA PM PGDP PHE Aging HC PPM, model(sem) emat(W) fe
```

```
est store eq_3
xsmle lnUHC GHE TA PM PGDP PHE Aging HC PPM, model(sem) emat(W) re
est store eq_4
xsmle lnUHC GHE TA PM PGDP PHE Aging HC PPM, model(sdm) wmat(W) ///
hausman effects fe
est store eq_5
xsmle lnUHC GHE TA PM PGDP PHE Aging HC PPM, model(sdm) wmat(W) ///
hausman re
est store eq_6

****LR test ****
lrtest eq_1 eq_5
lrtest eq_3 eq_5

****Hausman test ****
hausman eq_5 eq_6

local models " eq_1 eq_3 eq_5 eq_6 "
  ssc install estout
  esttab `models' using outcome37.csv ,append b(%9.5f) se(%6.3f) mtitle(`models') ///
        sca(r2 r2_w N F) star(* 0.1 ** 0.05 *** 0.01) nogaps

xsmle lnUHC GHE TA PM PGDP PHE Aging HC PPM, model(sdm) wmat(W) fe ///
type(both) nolog effects
est store both
xsmle lnUHC GHE TA PM PGDP PHE Aging HC PPM, model(sdm) wmat(W) fe ///
type(ind) nolog effects
est store ind
xsmle lnUHC GHE TA PM PGDP PHE Aging HC PPM, model(sdm) wmat(W) fe ///
type(time)
est store time

lrtest both ind ,df(31) stats
lrtest both time ,df(31) stats
lrtest time ind ,df(31) stats

local models " ind time both"
esttab `models' using outcome556.csv ,append b(%9.5f) se(%6.3f) mtitle(`models') ///
        sca(r2 r2_w N F) star(* 0.1 ** 0.05 *** 0.01) nogaps

****DSDM\\
xsmle lnUHC GHE TA PM PGDP PHE Aging HC PPM, wmat(W) model(sdm) dlag(1)
fe type(ind) durbin (GHE TA PM PGDP PHE Aging HC PPM) 0nsim(500) nolog
effects
```

```
outreg2 using 1111.doc
est store m1
local models "m1"
esttab "`m'" mtitle(`m1 ') nogap s(ar2 r2 sigma2 N ll aic bic) order(lnx1 lnx2 lnx3 lnx4)
esttab `models' using outcome26.csv ,append b(%9.5f) se(%6.3f) mtitle(`models')    ///
        sca(r2 r2_w N F) star(* 0.1 ** 0.05 *** 0.01) nogaps
```

Supplementary file 3. Supplementary output results

1. Spatial Autocorrelation Testing for UHC

Our study involved the computation of global spatial autocorrelation values for the Universal Health Coverage (UHC) index across diverse regions in China from 2004 to 2018. The Moran's index for UHC consistently exhibited a significant positive correlation at the 1% level for the majority of the years under study. Despite the Moran's index for UHC being negative in 2015, 2016, and 2018, it did not attain statistical significance. This implies that, UHC in China demonstrates a spatially positive correlation.

Table 1. Spatial Autocorrelation Testing for UHC

Year	Moran's I
2004	0.144***
2005	0.105***
2006	0.11***
2007	0.094***
2008	0.101***
2009	0.088***
2010	0.064***
2011	0.054***
2012	0.036***
2013	0.02*
2014	0.002
2015	-0.005
2016	-0.003
2017	0.003
2018	-0.001

Note: *, **, and *** indicate significance at the 10%, 5%, and 1% levels, respectively.

2. Spatial Panel Model Selection

We conducted LM tests to assess the feasibility of constructing a spatial model. As evident from the test results in Table 2, both the LM-error and Robust LM-error values of the model passed the test at a 1% significance level, indicating the suitability of selecting the Spatial Error Model (SEM). In this scenario, we proceeded to examine the applicability of the Spatial Durbin Model (SDM), conducting LR tests to assess whether it could be simplified to the SEM. The chi-square statistics for the Spatial Autoregression (SAR) and SEM were 73.34 and 104.29, respectively, both leading to the rejection of the null hypotheses (ho: the SDM can be simplified to the SAR, ho: SDM can be simplified to the SEM). This implies that SDM cannot be simplified into SEM. Therefore, our final choice for analysis was the SDM. In the context of spatial panel data, determining whether to employ fixed effects or random effects necessitates a Hausman test. We conducted Hausman test, comparing the consistency between fixed effects and random effects in the SDM, resulted in a chi-square statistic of -1.48. This

suggests that opting for fixed effects is more appropriate.

Table 2. Spatial Panel Model Selection Tests

Test	Chi-square statistic	P-value
Moran's I	4.026	0.0000
LM Error	243.803	0.0000
Robust LM Error	243.803	0.0000
LM lag	0.000	0.986
Robust LM lag	0.001	0.973
LR (SAR)	73.34	0.0000
LR (SEM)	104.29	0.0000
Hausman	-1.48	-
